# Cutaneous-Pericardial Fistula: Rare Complication of Transapical Aortic Valve Replacement—Case Report and Literature Review

**DOI:** 10.1155/2020/2371423

**Published:** 2020-07-22

**Authors:** Andrei Tarus, Mihail Enache, Igor Nedelciuc, Iulian Rotaru, Alberto Emanuel Bacusca, Grigore Tinica

**Affiliations:** ^1^Department of Cardiovascular Surgery, Cardiovascular Diseases Institute, Romania; ^2^“Grigore T. Popa” University of Medicine and Pharmacy, Iasi, Romania; ^3^Department of Interventional Cardiology, Cardiovascular Diseases Institute, Iasi, Romania; ^4^The Academy of Romanian Scientists (AOSR), Iasi, Romania

## Abstract

Cutaneous-pericardial fistula is a rare complication of transapical aortic valve replacement; only a few cases are reported in the literature. It is part of a wide range of surgical site infection manifestations that could emerge after surgery. Due to its proximity to the heart, the risk of infectious lesions of adjacent structures and inoculation of pathogens on the prosthetic valve can lead to life-threatening complications. We report here a case of successful surgical treatment through reduced ribs and soft tissue operative trauma.

## 1. Introduction

Since the first reported procedure in 2002, transcatheter aortic valve replacement (TAVR) has been changing the management of aortic stenosis, becoming the gold standard in the treatment of patients with prohibitive and high surgical risk [1]. TAVR became popular due to its mini-invasiveness and its wide range of technical approaches [2]. Despite the fact that TAVR proved to have better or at least comparable outcomes with conventional surgery [3, 4], the technique is still associated with numerous specific complications related to different implantation sites. Currently, the transfemoral approach is widely used, but not feasible in patients with severe peripheral arteries disease, unfavorable anatomy, or atherosclerotic aorta. Accordingly, transapical TAVR (TA-TAVR) proved to be a viable alternative in these cases despite its greater invasiveness [5]. TA-TAVR brought not only a new therapeutic solution for patients with aortic stenosis but also a new group of related complications. We report a case of TA-TAVR complicated by cutaneous-pericardial fistula at the wound site.

## 2. Case Report

A 55-year-old male with severe aortic stenosis and positive medical history for obesity (body mass index: 39.5 kg/m^2^), arterial hypertension, diabetes, prior coronary artery bypass grafting, and reduced left ventricular ejection fraction (3*5*%) underwent TAVR via the TA approach because of the severe iliac arteries stenosis. A 29 mm Edwards SAPIEN 3 valve (Edwards Lifesciences, Irvine, California) was successfully deployed.

Two pledget mattress sutures were used to close the insertion site. After protamine administration, additional BioGlue surgical adhesive (CryoLife, Inc., Kennesaw, GA) was used for complete hemostasis due to persistent mild bleeding. The pericardial and pleural cavity was drained.

The postoperative period was complicated by acute respiratory failure, acute kidney injury, and low cardiac output syndrome which necessitated prolonged inotropic support. The chest tubes were removed on the 8th postoperative day because of the persistent serous drainage. Two weeks after discharge, he developed aseptic wound seroma which was drained outside our service.

**Figure 1:**
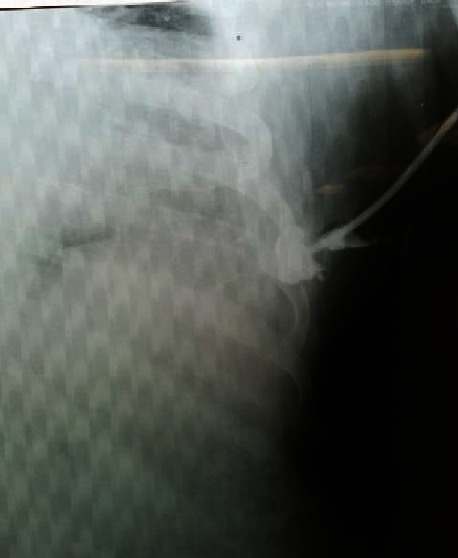
Fistulography of chronic draining sinus.

**Figure 2:**
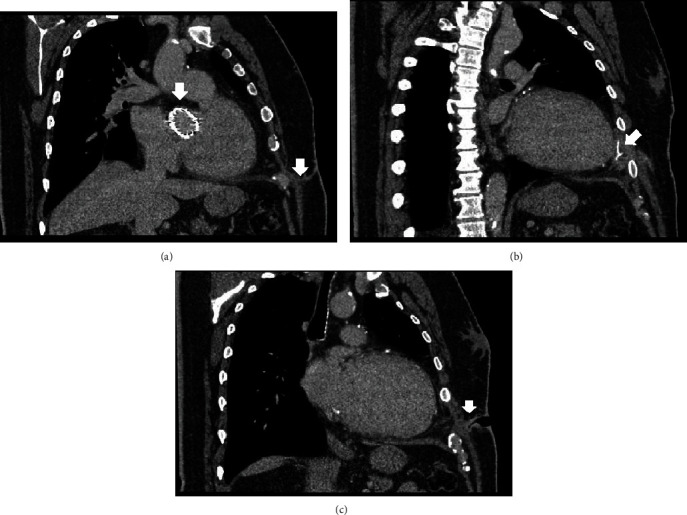
Thoracic computer tomography in (a) sagittal plane showing Edwards SAPIEN 3 prosthesis in aortic position and parafistulous soft tissue infiltration, (b) sagittal plane demonstrating para-apical collection and Teflon pledgets, and (c) sagittal plane presenting the “hourglass”-shape fistulous canal.

**Figure 3:**
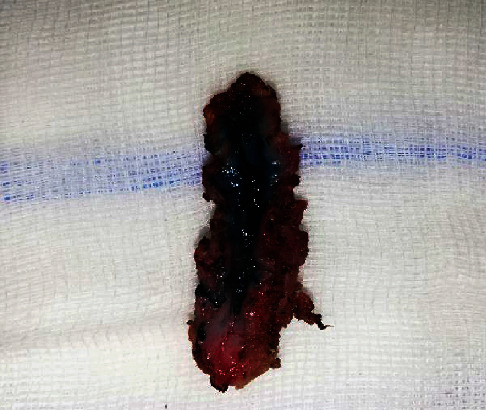
Chronic draining sinus.

Six months after discharge, he returned complaining about left chest pain and chronic draining sinus localized on the left thoracotomy scar. Blood counts showed normal leucocyte levels (6.51 × 103/*μ*l) and C-reactive protein (4.7 mg/l, reference range 0-5 mg/l). Microbiological culture tested negative. Fistulography revealed a cavity related to superficial layers of the thoracic wall (Figure 1). Thoracic computer tomography (CT) showed an oblique “hourglass”-shape fistula connecting the heart's apex and chest wall, a small apical collection (13 mm) with adjacent inflammatory infiltration involving pledget sutures (Figure 2). During surgery, the fistulous canal was marked with blue stains to prevent its opening and contamination of healthy tissues (Figure 3). The small aseptic abscess was incised near the heart apex; residual depots of BioGlue® (CryoLife, Inc., Kennesaw, GA) and pledget sutures were removed. The cavity was drained and the wound closed according to anatomic layers. Additional drainage was placed in the subcutaneous tissue. Long-term antibiotic treatment was performed with Vancomycin for bacterial endocarditis prophylaxis. Further evolution was uneventful. No recurrence was observed 12 months after discharge.

## 3. Discussion

Minimally invasive cardiac procedures were developed to reduce surgical trauma, thus diminishing related complications. Despite this, minithoracotomies are often associated with chronic pain and surgical site infection (SSI) [6, 7]. TA approach in TAVR proved itself a feasible solution, but with a group of rare and poorly studied complications [8–10].

Early SSI can trigger life-threatening bleeding or pseudoaneurysm formation [11]. Cutaneous-pericardial fistula after TA-TAVR can emerge as a late manifestation of the SSI. In most cases, it is a hospital-acquired infection and is classified as space/organ SSI according to the Centers for Disease Control criteria. To our knowledge, only 11 cases were reported in the literature (Table 1) [10, 12–15]. The mean age in the retrieved series was 80.4 ± 7.09 and the mean time from TAVR to the presentation was 5.4 months. In our case, the patient was much younger but with a lot of comorbidities, and the onset of clinical manifestation is included by the general trend.

The risk factors for SSI in cardiac surgery are well studied [16, 17]. Diabetes, obesity, chronic obstructive pulmonary disease (COPD), and respiratory failure are among the most important. In a TA-TAVR population, Baillot et al. reported an incidence of 3.2% of SSI, obesity being the most significant risk factor [13]. However, not only patient-related factors can influence the onset of SSI. Thus, Pasic et al. reported 3 cases of prolonged wound healing after BioGlue® (CryoLife, Inc., Kennesaw, GA) usage in TA-TAVR as a hemostatic agent [12]. In this particular case, additional hemostasis with BioGlue® (CryoLife, Inc., Kennesaw, GA) was necessary due to persistent mild bleeding after protamine administration. This surgical adhesive based on purified bovine serum albumin and glutaraldehyde is widely used in cardiac surgery, and its potential for inducing inflammation and tissue necrosis is well known [18]. Late aseptic reaction to this surgical adhesive was previously reported in cardiac and vascular surgery, manifested as a mediastinal cyst [19] and sterile abscess [20].

Microbiological profile in published cases included 4 patients with *Staphylococcus* spp., 3 negative cultures, and Gram-negative flora in 3 cases (*Enterobacter* spp., *Proteus* spp., and *Pseudomonas* spp.). Filsoufi et al. reported as causative agents *S. aureus* in 28 and *S. epidermidis* in 24 out of 106 patients with deep wound infection [16]. This means that pathogens are quite similar both in classic and minimally invasive approaches.

In reported cases, treatment strategy varied among different authors, from needle aspiration of liquid collection to complex technics with adjacent rib resection and reconstructions using pectoralis major muscles or great omentum. Baillot et al. advocated for a more aggressive approach without removing pledget material [13]. On the other hand, Pasic et al. performed local debridement and removed the pledgets [12], thus reducing surgical trauma, postoperative pain, and operation duration, factors which are essential during the recovery of high-risk patients. In our patient, rigorous excision of the fistulous canal after preventive blue stain marking, sanitation of residual cavity, extraction of pledget material, and wound drainage was enough for a recurrence-free result.

## 4. Conclusion

Cutaneous-pericardial fistula is a rare complication of TA-TAVR, which can be preceded by an extensive array of both intrinsic (obesity, diabetes, and COPD) and extrinsic (BioGlue usage) risk factors. The surgical approach is a treatment of choice for cutaneous-pericardial fistula with good postoperative results.

## Figures and Tables

**Table 1:** Summary of previously published cases.

Author	Case no.	Sex	Age	Onset post TAVI (months)	EF (%)	BMI, (kg/m^2^)	Comorbidities	Valve type	Valve size	Complications	BioGlue	Microbiology	Treatment	Outcome, follow-up
Pasic et al. [12]	1	F	87	3.8	60	23	—	ES	26	—	+	Sterile	Wound scar excision, remnants of BioGlue and pledgets removed, wound drainage+AB	No recurrence 21 months
	2	F	84	1.4	50	25	—	ES	23	—	+	*Pseudomonas fluorescens*	Wound scar excision, remnants of BioGlue and pledgets removed, wound drainage+AB	No recurrence 17 months
	3	F	87	2	50	28	—	ES	26	—	+	Skin flora	Wound scar excision, remnants of BioGlue and pledgets removed, wound drainage+AB	No recurrence 17 months

Baillot et al. [13]	4	F	79	24	65	33	TIA, hypothyroidism, CABG, CKD	ES	23	Pneumothorax UTI	−	*E. cloacae*	Wound debridement, rib resection, pectoralis major muscle flap+AB	—
	5	M	79	1	50	27	AF, COPD, gout, PVD, RA+steroids, DVT, CABG, CKD	ES	26	AF, renal failure, HF, pneumonia, empyema	−	*S. epidermidis*	Local debridement, fifth rib resection, empyema decortication, pectoralis major muscle flap+AB	—
	6	F	76	4	65	30	Hypothyroidism, DVT, PE, porcelain aorta	ES	23	AF/flutter	−	*S. epidermidis*	Local debridement, great omentum cover LV apex+AB	—
	7	F	64	2	50	35.2	DM, MI, PE, lung CA, RoRX	ES	26	HF	−	*S. aureus*	Local debridement, great omentum cover LV apex+AB	—
	8	F	77		20	31.2	PAF, PVD, COPD, active smoker	ES	26	HF	−	*S. epidermidis*	Wound debridement, rib resection, pectoralis major muscle flap+AB	—

Scheid et al. [10]	9	F	87	8	—	—	DM, HT, CABG	ES		Vacuum—assisted closure and antibiotics for wound infection	−	*Proteus mirabilis*	Surgical revision, pledgets removed, fifth and sixth rib resection+AB	No recurrence 3 months

Narala et al. [14]	10	M	77	4	—	—	CABG, recurrent IE, and chronic Q fever (hydroxychloroquine and doxycycline)	ES XT	29	Reexploration for hemorrhagic pericardial effusion, pneumonia, hematoma	−	Sterile	Rib resection, removal of the epicardial pacemaker lead, fistula repair, and replacement of the pledgets	—

Khan et al. [15]	11	F	87	4	38	—	HT, acute HF, pleural effusion	SAPIEN 3	23	—	−	Sterile	Needle aspiration	No recurrence 1 month

TIA: transitory ischemic attack; UTI: urinary tract infection; AF: atrial fibrillation; CA: cancer; CABG: coronary artery bypass grafting; CKD: chronic kidney disease; COPD: chronic obstructive pulmonary disease; PVD: peripheral vascular disease; DVT: deep venous thrombosis; DM: diabetes mellitus; MI: myocardial infarction; PAF: paroxysmal AF; PE: pulmonary emboli; RA: rheumatoid arthritis; RoRX: radiotherapy; HF: heart failure; IMV: invasive mechanical ventilation; GIB: gastrointestinal bleeding; SSI: surgical site infection; ES: Edwards SAPIEN; AB: antibiotics.
